# Novel therapeutics for coronary artery disease from genome-wide association study data

**DOI:** 10.1186/1755-8794-8-S2-S1

**Published:** 2015-05-29

**Authors:** Mani P Grover, Sara Ballouz, Kaavya A Mohanasundaram, Richard A George, Andrzej Goscinski, Tamsyn M Crowley, Craig D H Sherman, Merridee A Wouters

**Affiliations:** 1 School of Medicine, Deakin University, Geelong, Victoria, Australia; 2 Cold Spring Harbor Laboratory, Cold Spring Harbor, NY 11724, USA; 3 Victor Chang Cardiac Research Institute, 405 Liverpool St, Darlinghurst, 2010, NSW, Australia; 4 School of Information Technology, Faculty of Science Engineering and Built Environment, Deakin University, Geelong, Victoria, Australia; 5 Australian Animal Health Laboratory, CSIRO Biosecurity Flagship, Geelong, Victoria, Australia; 6 Life and Environmental Sciences, Deakin University, Geelong, Victoria, Australia

**Keywords:** Coronary Artery Disease, Genome-wide association study, Candidate gene, Drug database, Drug target, Drug repositioning

## Abstract

**Background:**

Coronary artery disease (CAD), one of the leading causes of death globally, is influenced by both environmental and genetic risk factors. Gene-centric genome-wide association studies (GWAS) involving cases and controls have been remarkably successful in identifying genetic loci contributing to CAD. Modern *in silico *platforms, such as candidate gene prediction tools, permit a systematic analysis of GWAS data to identify candidate genes for complex diseases like CAD. Subsequent integration of drug-target data from drug databases with the predicted candidate genes can potentially identify novel therapeutics suitable for repositioning towards treatment of CAD.

**Methods:**

Previously, we were able to predict 264 candidate genes and 104 potential therapeutic targets for CAD using *Gentrepid *(http://www.gentrepid.org), a candidate gene prediction platform with two bioinformatic modules to reanalyze Wellcome Trust Case-Control Consortium GWAS data. In an expanded study, using five bioinformatic modules on the same data, *Gentrepid *predicted 647 candidate genes and successfully replicated 55% of the candidate genes identified by the more powerful CARDIoGRAMplusC4D consortium meta-analysis. Hence, *Gentrepid *was capable of enhancing lower quality genotype-phenotype data, using an independent knowledgebase of existing biological data. Here, we used our methodology to integrate drug data from three drug databases: the Therapeutic Target Database, PharmGKB and Drug Bank, with the 647 candidate gene predictions from *Gentrepid*. We utilized known CAD targets, the scientific literature, existing drug data and the CARDIoGRAMplusC4D meta-analysis study as benchmarks to validate *Gentrepid *predictions for CAD.

**Results:**

Our analysis identified a total of 184 predicted candidate genes as novel therapeutic targets for CAD, and 981 novel therapeutics feasible for repositioning in clinical trials towards treatment of CAD. The benchmarks based on known CAD targets and the scientific literature showed that our results were significant (p < 0.05).

**Conclusions:**

We have demonstrated that available drugs may potentially be repositioned as novel therapeutics for the treatment of CAD. Drug repositioning can save valuable time and money spent on preclinical and phase I clinical studies.

## Background

Coronary artery disease (CAD) or Coronary heart disease (CHD) is a complex disorder which is a leading cause of death and disability (12.2%) worldwide [1]. In CAD, a waxy substance called 'plaque' collects inside the coronary arteries and other blood vessels which supply oxygen-rich blood to heart muscles [2]. Over time, hardened plaque narrows the coronary arteries, reducing the flow of oxygen-rich blood to the heart, resulting in CAD [2].

Environmental and genetic risk factors play an important role in the development of CAD. Lifestyle-related environmental factors include smoking, drinking and eating habits [3,4]. CAD is also inherited in families, suggesting the disease has a strong genetic basis [5]. CAD is thus a complex disease involving multiple risk factors, and is characterised by low penetrance of disease genes and non-Mendelian genetic transmission patterns. Heritability of CAD is estimated between 30%-60% by twin studies [6]. However, only a minor portion of heritability is explained by conventional risk factors such as decreased Low Density Lipoprotein (LDL) particle size and high Systolic Blood Pressure (SBP) [7,8].

Genome-Wide Association Studies (GWAS) are making progress towards revealing single-nucleotide polymorphisms (SNPs) associated with CAD. The Wellcome Trust Case Control Consortium (WTCCC) conducted the first large-scale GWAS study of 2,000 cases of CAD compared with white Europeans 3,000 controls [9]. The WTCCC study identified one highly independent association signal for CAD (p < 5 × 10^-7^) in the genetic locus 9p21 [9]. Another GWAS comparing 1,222 CAD cases with European 1,298 controls identified a second genetic locus (3q22) for CAD [10]. The typical effect sizes for individual SNPs were fairly small (~1%) in these studies.

In recent years, meta-analysis techniques have emerged as a successful approach for increasing the power of GWAS by pooling results from multiple GWAS studies. The Coronary ARtery DIsease Genome-Wide Replication and Meta-analysis (CARDIoGRAM) consortium identified 13 new genetic loci (p < 5 × 10^-8^) and 26 candidate genes in a meta-analysis study of 14 CAD GWASs comprising a total of 22,233 individuals with CAD compared to European 64,762 controls [11]. Another meta-analysis performed by the Coronary Artery Disease (C4D) Genetics Consortium identified five genetic loci for CAD (p < 5 × 10^-8^) and six candidate genes using data from four CAD GWAS comprising a total of 15,420 CAD cases (6,996 South Asians and 8,424 Europeans) and 15,062 controls (7,794 South Asians and 7,268 Europeans) which were replicated in an independent sample of 21,408 cases and European 19,185 controls [12]. Together, the CARDIoGRAM and C4D consortia (CARDIoGRAMplusC4D) scanned 63,746 CAD cases and 130,681 controls (South Asian and European) identifying 15 novel genetic loci and 20 likely candidate genes for CAD [13]. In total, these meta-analysis techniques successfully identified a further 32 genetic susceptibility loci for CAD beyond the two identified by the original studies. However, most of the identified genetic loci were limited to a highly significant statistical threshold (p < 5 × 10^-8^) because the genotype/phenotype data is inherently noisy.

Another approach to mining this inherently noisy data is to filter less statistically significant data using an independent data source. We previously developed protocols to predict candidate genes for complex diseases by reanalysing GWAS data using the *Gentrepid *candidate gene prediction tool as the biological knowledgebase, starting with data from a series of four lower statistical thresholds (p ≤ 5 × 10^-7^, p ≤ 10^-5^, p ≤ 10^-4^, p ≤ 10^-3^) [14]. *Gentrepid *utilizes five bioinformatic modules to predict candidate genes for complex diseases: two systems biology modules - Common Pathway Scanning (CPS) and Protein-Protein Interactions (PPI); one domain-homology recognition approach - Common Module Profiling (CMP) [14,15]; and two modules based on identification of nucleic acid regulatory factors involved in complex diseases - the common regulatory targets (CRT) module, and the microRNA regulatory module (MIR) [16]. Previously, we were able to predict 264 candidate genes for CAD [9,14] using two of these modules: CMP and CPS over six search spaces. In an expanded study, using a total of five bioinformatic modules: CMP, CPS, PPI, CRT and MIR [16], *Gentrepid *replicated 204 of the 264 predicted candidate genes in the previous two-module study, and identified an additional 443 candidate genes. In total, *Gentrepid *identified 647 candidate genes for CAD [16].

Compared to meta-analysis studies which have been performed for CAD, *Gentrepid *predicted 16%, 17% and 55% of the candidate genes identified in the CARDIoGRAM, C4D and CARDIoGRAMplusC4D meta-analysis studies respectively [11-13,16]. These data show that the *Gentrepid *results are in better alignment with the more powerful CARDIoGRAMplusC4D study which pooled cases and controls from the CARDIoGRAM and C4D studies.

Recently, we extended our computational pipeline by associating predicted candidate genes with drug-target information extracted from three publicly available drug databases: Drug Bank [17], the Pharmacogenomics Knowledgebase (PharmGKB) [18], and the Therapeutic Target Database (TTD) [19]. Applying this pipeline to the predicted candidate genes obtained by reanalysing WTCCC-GWAS data for seven complex diseases including CAD [14], we showed 38% of the predicted candidate genes (102 of 264 predicted candidate genes) are potential therapeutic targets for CAD, and predicted 743 novel therapeutics suitable for repositioning in clinical trials to accelerate the CAD drug discovery process [20].

In this study, we specifically focused on CAD, identifying novel therapeutic targets among the 647 predicted candidate genes for CAD by integrating drug-target association data extracted in the previous study [16,20]. We also identified novel therapeutic targets and associated novel therapeutics suitable for repositioning towards treatment of CAD. These were benchmarked using known CAD targets, the scientific literature, existing drug data and the CARDIoGRAMplusC4D meta-analysis study. We have demonstrated that it is possible to translate a large number of susceptibility genetic loci into clinical treatments of CAD using the *Gentrepid *candidate gene prediction tool. Thus, *Gentrepid *can be utilized as a drug discovery tool to identify novel treatments for CAD.

## Methods

**Figure 1:**
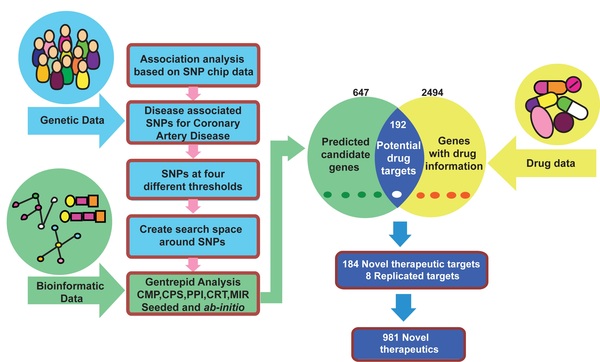
**Workflow**. Computational workflow to identify therapeutic targets and novel therapeutics for CAD by integrating genetic, bioinformatic and drug data. We used *Gentrepid *as a candidate gene prediction platform to predict candidate genes and DrugBank, TTD and PharmGKB as databases to extract drug data. Abbreviations - TTD - Therapeutic Target Database; PharmGKB - Pharmacogenomics Knowledgebase.

We implemented a workflow to identify potential therapeutics for CAD by integrating the two following data sets (Figure 1):

1. A predicted candidate gene data set for CAD, obtained by reanalysing the WTCCC-GWAS data [9], with *Gentrepid *using five bioinformatic modules: CMP, PPI, CPS, CRT and MIR [15,16];

2. A drug-gene target data set retrieved from three publically available drug databases namely TTD, DrugBank and PharmGKB [17,19,21].

### Candidate gene data set

In our previous work, we predicted a total of 647 candidate genes for CAD by careful reanalysis of the WTCCC GWAS data on CAD [9] using the *Gentrepid *candidate gene prediction system [16].

The WTCCC study used a highly stringent significance threshold (p ≤ 5 × 10^-7^) to correct for multiple testing in GWAS analysis [9]. While robust, this approach resulted in association of only one genetic locus with CAD [14]. To address the high false negative rate of GWAS studies, we previously proposed a bioinformatics strategy to sift through genes near the implicated loci of a large number of SNPs of slightly lower significance thresholds. We considered four thresholds of decreasing stringency: a highly significant set (HS - p ≤ 5 × 10^-7^), a Medium highly significant set (MHS - p ≤ 10^-5^), a Medium weakly significant set (MWS - p ≤ 10^-4^), and a weakly significant set (WS - p ≤ 10^-3^). In total, we constructed a series of four SNP sub sets comprising a total of 757 SNPs for CAD [14].

An additional problem arises when mapping these SNPs to nearby genes. Although the causal SNPs are likely to be in linkage disequilibrium with the implicated SNPs, genomic architecture is still not well understood. The implicated SNP may be in a control region distal to the transcribed region of the gene. Six different search spaces - three of fixed-widths and three proximity-based, were created around each SNP-based genetic locus, for analysis by the *Gentrepid *candidate gene prediction system [14]. Thus, we utilized six gene selection methods around each SNP to construct the gene search spaces, using four SNP sets acquired by incrementally lowering the significance threshold of the data, resulting in a total of 24 search spaces [9,14].

For each of these 24 search spaces, we used the following five bioinformatic modules to predict and prioritize candidate genes for CAD using the *Gentrepid *candidate gene prediction tool [16]: Two systems biology approaches - a) Common Pathway Scanning (CPS) and, b) Protein-Protein interaction module (PPI); one domain homology module - c) Common Module Profiling (CMP) and; two nucleic acid based regulatory modules - d) Common Regulatory Targets (CRT) and, e) the Micro-RNA regulatory module (MIR) [16].

The two systems biology modules, CPS and PPI, are based on the principle that common phenotypes are associated with proteins that participate in the same protein complex or biochemical pathway [22]. The domain-homology module, CMP, is a sequence analysis approach based on the assumption that candidate genes are similar in function to disease genes already determined for the phenotype [23]. We have described these methods in detail in our previously published work [14,15,20].

The two nucleic acid-based regulatory modules: CRT and MIR are based on the assumption that disruption of regulatory elements controlling gene expression can cause diseases [24]. CRT searches for genes in the susceptibility genetic loci that bind with common transcription factors. Regulatory information for genes of the search space was retrieved from the Open REGulatory ANNOtation (oRegAnno) database, a publically available database of curated known regulatory elements from the scientific literature [25]. The MIR module is based on the assumption that dysfunction of micro-RNAs (miRNAs) plays a key role in the heart, central nervous system, and immune system-related diseases [26]. MIR searches the genetic susceptibility loci for genes which are common miRNA targets and present in regulatory hubs [16]. MicroRNA information for this module was extracted from the mirBase database, an online repository for microRNA sequences and annotations [27].

### Drug-gene target data set

We used a drug-gene target data set compiled from three online drug databases: DrugBank [17], PharmGKB [21] and TTD [19], described in detail in our previously published work [20].

DrugBank is a chemical and clinical drug database [18], combining detailed drug data and disease information with comprehensive drug-target associations [17]. Previously, we retrieved 6,711 drug entries active against 3,410 unique drug targets for several species from DrugBank [20]. We used the G-profiler conversion tool to translate human drug target information to official HUGO gene symbols [20,28], resulting in a dataset comprising 3,910 drugs associated with 2,022 human drug targets [20].

The Pharmacogenomics Knowledgebase (PharmGKB) is a clinical drug database, combining information about drugs, diseases and targeted genes [21]. This database describes around 3,097 drugs and 26,961 human genes, but not all of these genes are associated with drugs. We obtained a licensed PharmGKB annotation dataset, describing a total of 382 drugs associated with 566 human drug targets [20].

The Therapeutic Target Database (TTD) is a chemical drug database, integrating drug data with therapeutic targets [19]. TTD contains 17,816 drugs (approved, clinical and experimental) associated with 2,025 human and non-human drug targets. We replaced the UniProt accession numbers with official HUGO gene symbols using the G-profiler conversion tool [28], extracting 2,960 drugs for 544 unique human drug targets [20].

Pooling the data from DrugBank, TTD and PharmGKB, we obtained a total of 2,494 unique gene targets from all the databases, comprising ~ 8% of the entire human genome [20]. A comparison of the extracted drug-target datasets from the three databases revealed that only 4% of human drug targets were common to all three drug databases [20]. We retrieved the maximum number of unique targets from DrugBank (1,495), followed by TTD (129), and PharmGKB (326) [20]. In pairwise comparisons, DrugBank and TTD share the maximal number of drug targets (398), while TTD and PharmGKB share the fewest (111) [20].

Of the 9,991 unique drugs contained in these three drug databases [20], 50% of them are found only in DrugBank, while the unique contributions from TTD and PharmGKB were 15-18% [20]. In pairwise comparisons, TTD and PharmGKB share 15-19% of their retrieved drugs with DrugBank [20]. DrugBank and PharmGKB share the maximal number of drugs (1620), while TTD and PharmGKB share the fewest (1352) [20]. In total, we retrieved a total of 7,252 unique drugs associated with 2,494 human drug targets from all three drug databases [20].

### Identification of novel therapeutics and therapeutic targets

We mapped predicted therapeutic targets from the predicted candidate genes with the extracted drug-gene target association files. A total of 647 predicted candidate genes for CAD were mapped separately with the three drug-target association files, and results were retrieved.

Within this set, we distinguished known and novel therapeutic targets and therapeutics for CAD. If a drug associated with a therapeutic target is not registered as a therapy for CAD, it is designated as a novel therapeutic directed towards a predicted candidate gene target for CAD. Novel therapeutics may be suitable for repositioning towards treatment of CAD.

### Comparison with previous studies

We compared therapeutic targets obtained in our previous study with this study to identify therapeutic targets for CAD using the WTCCC-GWAS data. In our previous study, we utilised the CMP and CPS bioinformatic modules to predict candidate genes and therapeutic targets [14,20]. In this study, we integrated the results from five bioinformatic modules: CMP, PPI, CPS, CRT and MIR. Thus, we compared therapeutic targets obtained from two different bioinformatic studies conducted to reanalyse the same WTCCC-GWAS data [9].

The CARDIoGRAMplusC4D consortium meta-analysis compared 63,746 CAD cases with 130,681 controls identifying 15 genetic loci and 20 candidate genes for CAD [29]. Our previous reanalysis of this data with *Gentrepid *replicated 11 of the 20 candidate genes and made three novel gene predictions (*LRPPRC, GUCY1B3, MAP3K4*) [16]. In this study, we identified potential therapeutic targets after mapping the 20 candidate genes obtained from the CARDIoGRAMplusC4D study data and the three novel genes predicted by *Gentrepid *with the extracted drug-gene target dataset. We also compared the identified therapeutic targets from the CARDIoGRAMplusC4D study with the *Gentrepid*-predicted therapeutic targets.

### Validation of predicted therapeutic targets

We validated the predicted therapeutic targets using two benchmarks as described in our previously published work [20]. The first benchmark tested the ability of *Gentrepid *to replicate known therapeutics for CAD. However, this benchmark does not give any idea about the validity of the novel predictions for CAD. Therefore, we performed a second benchmark to assess the validity of the candidate gene predictions using text mining of the existing Pubmed literature for CAD.

In the first benchmark, we classified genes present in the six search spaces as "CAD candidates" or "CAD non-candidates". We considered genes which are already known drug targets for CAD as "true positives". Targets which were not predicted by *Gentrepid*, but present in the search space and targeted by currently registered therapeutics for the CAD, were designated "false negatives". Genes, which were neither predicted for CAD nor targetable by CAD drugs, were designated as "true negatives"; and predicted novel therapeutic targets were selected as "false positives". Finally, we plotted a Receiver Operation Characteristic (ROC) curve considering six thresholds based on the number of targets present in the six search spaces constructed (see candidate gene dataset section in *Method*s for details). Non-linear regression analysis, was performed to fit the ROC curves (see Validation of predicted therapeutic targets in *Results and Discussion*).

In the second benchmark, we extracted Pubmed IDs of literature related to CAD from Pubmed in Feb. 2014. We mapped the retrieved Pubmed IDs to the gene citation data downloaded from Entrez Gene (ftp//http://ftp.ncbi.nih.gov/gene/) to calculate the number of article citations for each target, using both the gene name and the phenotype name (CAD). Further, a ROC curve was created considering four thresholds of article citations (one, five, ten and fifteen). Finally, non-linear regression analysis was performed to fit the ROC curve (see Validation of predicted therapeutic targets in *Results and Discussion*).

## Results and Discussion

### Discovery of novel therapeutic targets

*Gentrepid *identified 647 candidate disease genes for CAD [16]. We searched for potential drug-targets in the extracted drug gene-target files from the three drug databases and found 192 candidate genes (30%) are potential therapeutic targets for CAD (Figure 1). This may seem like a large number, but as the typical effect sizes of the most significant loci in the original WTCCC study was ~1%, and the estimated heritability of CAD is 30-60%, a minimum of 30-60 genes are expected to underline the disease. Therefore, it is not implausible that all of these predicted genes are involved in aetiology of CAD.

**Figure 2:**
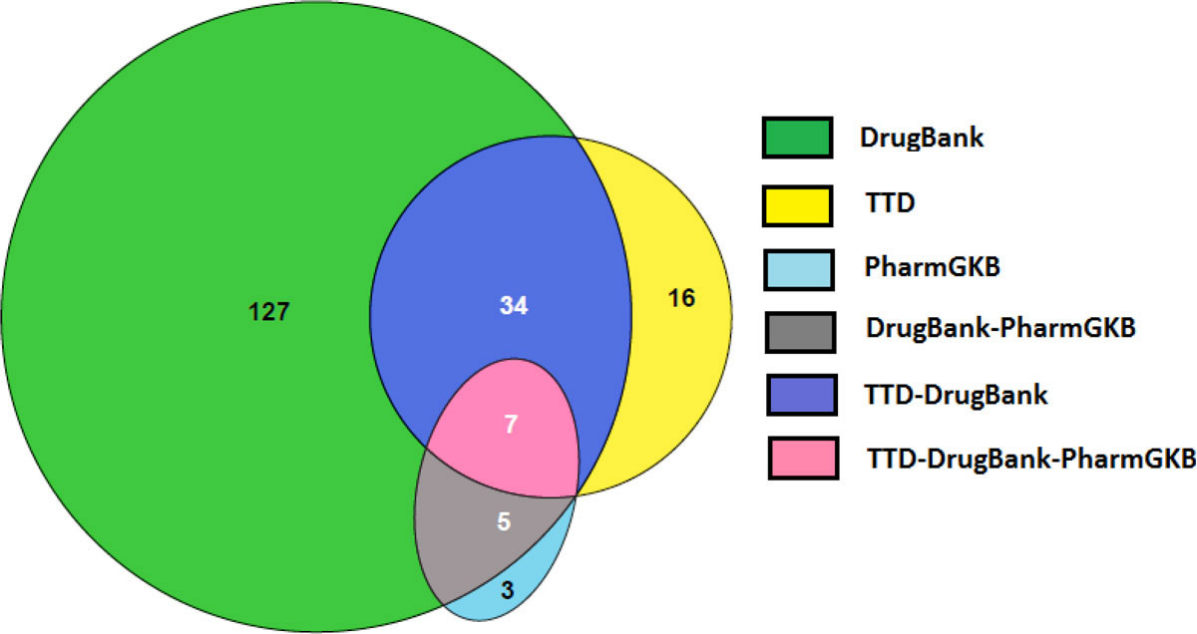
**Predicted therapeutic targets for CAD by drug database**. Therapeutic targets for CAD obtained from three drug databases. The maximum contribution was from DrugBank (173). A further 19 unique targets were contributed by TTD and PharmGKB. We also identified seven therapeutic targets common to all three drug databases. In pairwise comparisons, there were no common therapeutic targets between the TTD and PharmGKB databases that were unique to these two databases i.e. not found in DrugBank. However, there were 34 targets common to TTD and DrugBank, and five targets common to PharmGKB and DrugBank databases. Abbreviations - CAD - Coronary Artery Disease; TTD - Therapeutic Target Database, PharmGKB - Pharmacogenomics Knowledgebase.

Each drug database made significant contributions to therapeutic target identification, with the maximum contribution from DrugBank (173), followed by TTD (57) and PharmGKB (15) (Figure 2). The enrichment of druggable targets in the predicted candidate gene dataset for CAD was 30% compared to the value of ~8% for the entire genome which might be a selection effect, either at the genome level or the knowledgebase level [20]. For instance, at the genome level, it has been posited that a set of "troublemaker" or disease genes exists [30]. Alternatively, at the knowledgebase level, we may know more about drugs for the CAD phenotype-associated genes as a subset of genes in the human genome, than the remainder of the genes in the genome.

We performed a binary classification of the 192 predicted therapeutic targets to distinguish novel and replicated therapeutic targets. Novel therapeutic targets are genes targeted by therapeutics already approved, or still in clinical trials for other diseases, but not for CAD. We found 184 novel therapeutic targets, accounting for almost 95% of the targets identified in our analysis. A selection of these are shown in Table 1. The remaining eight targets have therapeutics which are either approved or in ongoing clinical trials for CAD (Table 2). These eight targets are *Gentrepid-*predicted therapeutic targets that are already known to be associated with CAD (Table 1), and are thus replicated directly from the genetic data *de novo*. These eight replicated known targets are designated "true positives" in the first benchmark described below. We also identified 30 known targets of drugs used in the treatment of CAD present in drug databases, which were not predicted by *Gentrepid *from the WTCCC GWAS data (Additional file 1 - Table S1). Most of these were not present in the search spaces constructed from the genetic data: suggesting that the genetic data is at odds with these currently used therapeutics; or the genetic architecture is more complicated than was assumed during construction of the search spaces. However, four of these 30 targets are present in all six of the search spaces constructed for the weakly significant dataset, but were not retrieved by *Gentrepid *(Additional file 1 - Table S1). This may be failure of the system at the knowledgebase level, possibly due to incomplete coverage by the databases used. These four targets are considered false negatives in the first benchmark described below (Validation of predicted therapeutic targets in *Results and discussion*).

**Table 1:** Selected novel therapeutics suitable for repositioning for CAD

Target	*Drug name	Disease	Action	Status	*Database
*CHRM3*	Tiotropium	Chronic obstructive pulmonary disease	Antagonist	Approved	TTD
*HTR1A*	Fluvoxamine	Depressive disorder	Unknown	Unknown	PharmGKB
*FLT1*	Sorafenib	Advanced renal cell carcinoma	Inhibitor	Launched	TTD
*ABAT*	Vigabatrin	Epilepsy	Inhibitor	Approved	TTD
*GRIK2*	Metharbital	Epilepsy	Antagonist	Approved	DrugBank
*IL2RB*	Aldesleukin	Metastatic renal cell carcinoma	Agonist	Approved	DrugBank
*ITGB1*	Antithymocyte globulin	Prevention of renal transplant rejection	Unknown	Approved	DrugBank
*PDGFRA*	Becaplermin	Skin ulcers (from diabetes)	Unknown	Approved	DrugBank
*IL2RB*	Daclizumab	Prevention of renal transplant rejection	Antibody	Approved	DrugBank
*VEGFA*	Bevacizumab	Metastatic breast cancer	Unknown	Approved	DrugBank

Selected novel therapeutics suitable for repositioning to develop potential treatment of CAD.

(* These drugs mentioned here are only selected examples because one therapeutic target may be associated with multiple drugs); (* Drug databases mentioned here are only selected examples because one drug-target association may be described in more than one drug database). Abbreviations - PH - Phenotype; TTD - Therapeutic Target Database; PharmGKB - Pharmacogenomics Knowledgebase; CAD - Coronary Artery Disease.

**Table 2:** Replicated therapeutics for CAD

Target	*Drug name	Status	Action	*Database
*PLG*	Anistreplase	Approved	Activator	TTD
*ALOX5AP*	DG031	Suspended in Phase III	Inhibitor	TTD
*PLAT*	Urokinase	Approved	Activator	DrugBank
*AGTR1*	Losartan	Approved	Antagonist	DrugBank
*NOS3*	ACCLAIM	Phase III	Unknown	DrugBank
*PLAUR*	Urokinase	Approved	Activator	DrugBank
*NID1*	Urokinase	Approved	Unknown	DrugBank
*MYC*	AVI4126	Phase I/II	Antisense	TTD

Eight therapeutic targets with examples of replicated known therapeutics for CAD in this study.

* Drugs shown are examples. More than one therapeutic drug may be associated with each replicated target; * Drug databases shown are examples. One drug-target association may be described in more than one drug database. Abbreviations - PH - Phenotype; CAD - Coronary Artery Disease; TTD - Therapeutic Target Database.

**Figure 3:**
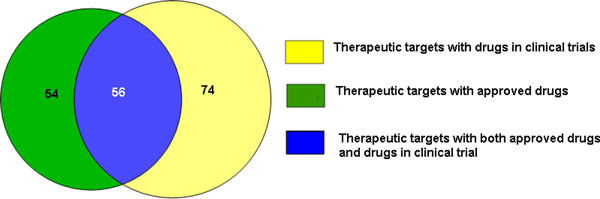
**Novel therapeutic targets with approved drugs compared to targets with drugs in clinical trials**. A total of 110 therapeutic targets with approved drugs were identified which may benefit the CAD phenotype, with a further 74 novel therapeutic targets in clinical trials. Therapeutic drugs in the overlapping set are approved for one phenotype, and also in clinical trials for a second phenotype.

We further classified the novel targets into targets of approved drugs vs targets of drugs in clinical trials. We found 53 targets with approved drugs, 74 targets with drugs in clinical trials, and 56 targets of both approved drugs and drugs in clinical trials (Figure 3). Both approved drugs, and drugs in clinical trials associated with the novel targets, are suitable for repositioning towards treatment of CAD. However, approved therapeutics associated with novel targets will be the priority for further repositioning studies because of the lower risk involved.

### Identification of novel therapeutics

We identified novel therapeutics by comparing indications of predicted drugs with our phenotype of interest i.e. CAD. If a drug is neither approved nor in clinical trial for CAD, it is predicted as a novel therapeutic suitable for repositioning in clinical studies. Of the 993 identified unique drugs, we found the maximum number of drugs from DrugBank (821), and the remainder from TTD (234) and PharmGKB (23). By comparing the indications of predicted drugs with the phenotype (CAD), we determined 981 of the 993 predicted drugs are novel therapeutics. The percentage of drugs that may be repositioned towards treatment of CAD was around 14% of the total number of drugs extracted from the databases (981 of 7,252 extracted drugs). In total, we found 981 novel therapeutics: 331 of these were approved, 636 were in clinical trials, and 14 were both approved and in clinical trials for diseases other than CAD. For example, the drug succinylcholine, which acts upon the *CHRM3 *gene product, is approved as a therapeutic for spasm (Table 1). Our study predicts *CHRM3 *as a predicted candidate gene and novel therapeutic target for CAD, suggesting that the drug succinylcholine may be repositioned as a novel therapeutic for CAD.

### Identification of known therapeutics

We replicated 12 known therapeutics for the eight *Gentrepid*-replicated targets for CAD (Table 2). For example, the approved drug anistreplase, retrieved from the TTD database, targets plasminogen, *PLG*: a predicted therapeutic target for CAD. Losartan, an antagonist of the type 1 angiotensin receptor, *AGTR1*, is another known CAD therapeutic retrieved from DrugBank (Table 2). Thus, the system is capable of replicating known therapeutics for CAD directly from the genetic data.

### Validation of predicted therapeutic targets

We used two different benchmarks to assess the validity of targets predicted by *Gentrepid *for CAD. In the first benchmark, we validated association of targets with CAD based on whether they are designated as known targets for CAD in the drug databases or not. This was performed for all six search spaces created (see *Methods *for details). In the second benchmark, we retrieved the number of Pubmed citations, citing both the phenotype of interest (CAD) and the gene name to validate the association of the predicted gene-target with CAD.

For the first benchmark, we classified genes in the six search spaces as "CAD candidates" or "CAD non-candidates". Targets with known therapeutic drugs for CAD were considered "true positives" (Table 3). Targets which were not predicted by *Gentrepid*, but present in any of the six search spaces, and targeted by currently registered therapeutics for CAD were considered as "false negatives". Genes that were not predicted for CAD and not targetable by CAD drugs were regarded as "true negatives", while *Gentrepid*-predicted novel therapeutic targets for CAD were considered "false positives" (Table 3). A ROC curve was plotted considering targets present in the six search spaces constructed for the weakly significant data set (Table 3 Additional file 1 - Figure S1(A)). The Area Under Curve (AUC) value of these ROC curves was greater than 0.5 (p < 0.05) suggesting that our predictions of therapeutic targets for CAD are significant (Additional file 1 - Figure S1(A)).

**Table 3:** Binary classification of targets present in six search spaces

	Known Drug Targets	Novel Drug Targets
**CAD candidates**	T.P. = 8	F.P. = 184
**CAD non-candidates**	F.N. = 4	T.N. = 4,519

**Σ 4,715**		

Binary classification of therapeutic targets considering six thresholds based on therapeutic targets present in six search spaces constructed in weakly significant data set (WS). Abbreviations: CAD - Coronary Artery Disease; TP - True Positives; FP - False Positives; TN - True Negatives; FN - False Negatives.

For the second benchmark, a ROC curve for CAD was created by considering four thresholds for targets with at least one, five, ten and fifteen Pubmed citations as CAD true positives and targets with less than five, ten and fifteen citations or without any citations as CAD false positives (Additional file 1 - Figure S1(B)). Genes with at least one, five, ten and fifteen article citations not predicted by *Gentrepid*, but present in the search space were considered as "false negatives". Genes neither cited nor predicted for CAD were regarded as "true negatives". The AUC value for this ROC curve was also significantly greater than 0.5 (p < 0.05) ensuring that our results are not generated by chance and our predictions of therapeutic targets for CAD are significant (Additional file 1 - Figure S1(B)).

### Comparison with previous studies

**Figure 4:**
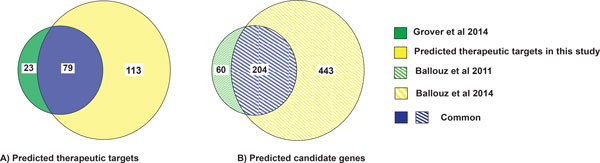
**Comparison of therapeutic targets and candidate genes obtained after reanalysing WTCCC-GWAS data in 5-module study vs our previous 2-module study**. A) Comparison of therapeutic targets for CAD obtained in the two different studies by utilizing different number of bioinforamtic databases to reanalyse the same WTCCC-GWAS data. In this study, 79 therapeutic targets were replicated from the previous study (Grover et al, 2014), and 113 additional therapeutic targets were identified; B) Comparison of predicted candidate genes obtained by reanalysing the same WTCCC-GWAS data in the two different studies (Green crosshatched portion - Ballouz et al, 2011, Yellow crosshatched portion - Ballouz et al, 2014). In our recently published study for CAD (Ballouz et al 2014), 204 candidate genes were replicated from the previous study (Ballouz et al, 2011) and 443 additional candidate genes were identified (Ballouz et al, 2014). Abbreviation - CAD: Coronary Artery Disease.

In our previous bioinformatic analysis of the WTCCC-GWAS data, we identified 102 of 264 predicted candidate genes as therapeutic targets for CAD using only the CMP and CPS modules [20]. In this study, we identified 192 of 647 *Gentrepid*-predicted candidate genes of CAD as therapeutic targets using a total of five bioinformatic modules - CMP, PPI, CPS, CRT and MIR. We compared the therapeutic targets obtained for CAD in both studies and observed that more than half (59%) of the therapeutic targets were not identified previously (Figure 4A). In total, 113 therapeutic targets were not identified in our earlier study, and 79 therapeutic targets are common to both studies (Figure 4A).

We also sought to understand how druggable the candidate genes predicted in the newer study, based on 5 bioinformatic modules, were compared to our older 2-module study. Among the 647 candidate genes utilised in this study, 204 candidates were replicated from our previous study. We calculated a Targetability Index (TI), the ratio of predicted therapeutic targets to predicted candidate genes. Although 443 additional candidate genes were predicted compared to our previous study, the proportion of these that mapped to therapeutic targets, TI_5, _was lower (30%, n = 192) for the 5-module study compared to the value, TI_2_, for the 2-module study, (39%, n = 102) [20]. This is likely a selection effect due to better knowledge of genes in pathway databases compared to those modules based on the high throughput data (PPI, CRT, MIR).

The novelty of the predicted therapeutic targets for CAD was also compared between this study and our previous study. A novelty ratio was calculated as the ratio of number of novel therapeutic targets i.e. those that have not been previously associated with CAD, to the number of predicted therapeutic targets for CAD [20]. The novelty ratio in this 5-module study for CAD was 0.95 (184/192) roughly the same as our previous 2-module study (0.96) (98/102) [20]. This suggests that the relative number of repositioning opportunities did not decrease, despite the yield of therapeutic targets going down when the additional bioinformatic modules were added, as indicated by the TI [20].

**Figure 5:**
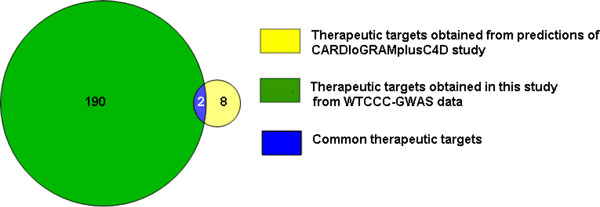
**Comparison of *Gentrepid *predicted therapeutic targets based on WTCCC-GWAS data vs CARDIoGRAMplusC4D study data**. Comparison of therapeutic targets predicted from CARDIoGRAMplusC4D study data with predicted therapeutic targets from WTCCC-GWAS data. The system identified two (*PLG and FLT1*) of ten therapeutic targets obtained from CARDIoGRAMplusC4D study data.

We also compared our results with the most powerful meta-analysis of CAD which has yet been performed. The CARDIoGRAMplusC4D study improved the statistical power of the genetic analysis by increasing the number of cases and controls by a factor of 10 over the original WTCCC study. We mapped the 20 candidate genes for CAD obtained in the CARDIoGRAMplusC4D study with the extracted drug-gene target dataset and identified ten therapeutic targets. *Gentrepid *independently predicted two (*PLG *and *FLT1*) of the ten therapeutic targets obtained using the CARDIoGRAMplusC4D study data (Figure 5). Thus, the *Gentrepid *system was able to successfully retrieve 20% of the therapeutic targets obtained using the CARDIoGRAMplusC4D study data. To summarize, our analysis showed that the system not only replicated already known targets, but also made novel valid predictions using existing biological and drug knowledgebases.

## Limitations

Although *Gentrepid *was able to predict a list of 981 potential novel therapeutics suitable for repositioning towards treatment of CAD, further clinical trials are required to confirm the efficacy of these novel therapeutics. Repositioning opportunities will not always be successful due to the complexity, variability and sparsity of currently available data in the biological knowledge bases, and to the intrinsic nature of genetic data [31]. However, one successfully repurposed drug can significantly impact the drug development for a complex disease [31]. The results presented here can accelerate drug discovery programs for CAD by translation of already known compounds for novel therapeutic uses towards CAD. Overall, our pipeline is an appropriate methodology for generating potential therapeutics for CAD from GWAS data.

## Conclusion

CAD is a complex trait that has a major impact on human morbidity and mortality. Identification of potential therapeutic targets is necessary to develop novel treatments for complex diseases like CAD. In this study, we integrated known drug data with predicted candidate genes for CAD. We found 30% (n = 184) of the predicted candidate genes could serve as novel therapeutic targets, and 14% (n = 981) of the retrieved drugs are potential novel therapeutics for CAD. Novel therapeutics include both FDA-approved drugs and drugs currently in clinical trials. Hence, these drugs may be repositioned towards treatment of CAD. The lower effect sizes of individual loci and large number of predicted targets suggest that cocktails of repositioned drugs may be therapeutically effective. Thus, *Gentrepid *offers new directions in repositioning of already known drugs to discover novel-cost effective treatments for CAD.

## List of abbreviations

CAD: Coronary Artery Disease; CHD: Coronary Heart Disease; GWAS: Genome-Wide Association Study; SNP: Single Nucleotide Polymorphism; TTD: Therapeutic Target Database; PharmGKB: Pharmacogenomics Knowledgebase; FDA: Food and Drug Administration; CPS: Common Pathway Scanning; CMP: Common Module Profiling; WTCCC: Wellcome Trust Case-Control Consortium; AUC: Area Under Curve; TI: Targetability Index; ROC: Receiver Operation Characteristic curve; WS: Weakly Significant set; MWS: Moderately-Weak Significant set; MHS: Moderately-High Significant set; HS: Highly Significant set.

## Competing interests

The authors declare that they have no competing interests.

## Authors' contributions

MPG worked on the design of the project and also performed data mining and data analysis. MAW conceived the project and reviewed the results obtained after data analysis. MPG, MAW, TMC, KAM, CDS, SB, RAG and AG helped to draft the manuscript. All authors read and approved the final manuscript.

## Supplementary Material

Additional file 1 Known targets and ROC curves for CAD. Table S1 - List of 30 known targets of CAD retrieved from drug databases, not predicted by *Gentrepid*. Four of these 30 known targets of CAD are present in all of the six search spaces. Abbreviations - ROC - Receiver Operation Characteristics Curve; AUC - Area Under Curve. Figure S1 - A) ROC curve for CAD based on six thresholds obtained from targets present in six search spaces in weakly significant data set (WS) (AUC - 1.0). B) ROC curve for CAD based on four thresholds obtained using four cut-off of Pubmed citations (at least one, five, ten and fifteen) (AUC - 1.0). Abbreviations - ROC - Receiver Operation Characteristics Curve; AUC - Area Under Curve. Click here for file

## Declarations

The publication costs for this article were funded by the School of Medicine, Deakin University, Australia.

This article has been published as part of *BMC Medical Genomics *Volume 8 Supplement 2, 2015: Selected articles from the 4th Translational Bioinformatics Conference and the 8th International Conference on Systems Biology (TBC/ISB 2014). The full contents of the supplement are available online at http://www.biomedcentral.com/bmcmedgenomics/supplements/8/S2.
