# Supplementary material for: Novel therapeutics for coronary artery disease from genome-wide association study data
Source: BMC Med Genomics. 2015 May 29;8(Suppl 2):S1. doi: 10.1186/1755-8794-8-S2-S1 (PMC4460746; doi:10.1186/1755-8794-8-S2-S1)
Supplement: Additional file 1 — Known targets and ROC curves for CAD. Table S1 - List of 30 known targets of CAD retrieved from drug databases, not predicted by Gentrepid. Four of these 30 known targets of CAD are present in all of the six search spaces. Abbreviations - ROC - Receiver Operation Characteristics Curve; AUC - Area Under Curve. Figure S1 - A) ROC curve for CAD based on six thresholds obtained from targets present in six search spaces in weakly significant data set (WS) (AUC - 1.0). B) ROC curve for CAD based on four thresholds obtained using four cut-off of Pubmed citations (at least one, five, ten and fifteen) (AUC - 1.0). Abbreviations - ROC - Receiver Operation Characteristics Curve; AUC - Area Under Curve. [file 1755-8794-8-S2-S1-S1.pdf]

Table S1 – Known targets of CAD

Known targets of CAD not predicted by *Gentrepid*	Known targets of CAD present in six search spaces, not predicted by *Gentrepid*
CYP2C19	SERPINA5
P2RY12	GUCY1A2
ABCB11	P2RY12
PLA2G2A	PCSK9
MAPK12	
F9	
PCSK9	
ALOX5	
NPR1	
GUCY1A2	
ADRB1	
SERPINC1	
PRSS1	
PLAU	
SERPINE1	
SERPINB2	
SERPINA5	
LRP2	
ST14	
ADRB2	
ADRB3	
F10	
SELP	
CTRB1	
KLK1	
HMGCR	
ITGAL	
HDAC2	
DPP4	
AHR	

Figure S1 - ROC curves

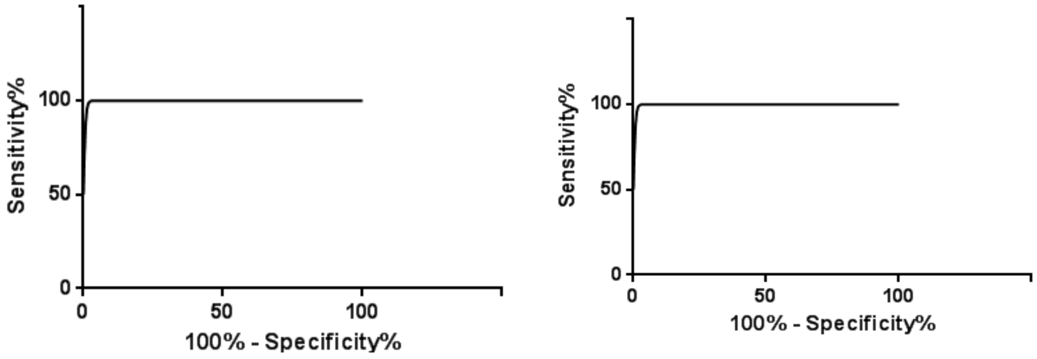

A **B**
